# A Study on the Heat Transfer Characteristics of Steel Plate in the Matrix Laminar Cooling Process

**DOI:** 10.3390/ma14195680

**Published:** 2021-09-29

**Authors:** Jing Xu, Guang Chen, Xiangjun Bao, Xin He, Qingyue Duan

**Affiliations:** 1 School of Metallurgical Engineering, Anhui University of Technology, Ma’anshan 243032, China; shiliu1109@126.com; 2 School of Energy and Environment, Anhui University of Technology, Ma’anshan 243032, China; baoahut212@163.com (X.B.); Hexin123456782021@163.com (X.H.); 3 School of Mechanical Engineering, Wanjiang University of Technology, Ma’anshan 243031, China; Dqy20000212@163.com

**Keywords:** laminar cooling, cooling rate, surface integrated heat transfer coefficient

## Abstract

Accurate prediction and control of the steel plate temperature in the laminar cooling process are very challenging. In this research, an experimental platform was built to measure the heat transfer characteristics of the steel plate in the process of matrix laminar spray cooling when the steel plate is one millimeter away from the upper surface. The “buried couple method” was used, including the cooling temperature and cooling rate. Then, the temperature and the integrated heat transfer coefficient at the steel plate surface were calculated by the time-sequential function method (TSFM). The obtained results show that the fast cooling stage under the water cooling condition occurred in the first 1.5 s, and the measuring point temperature decreased by 8%. The “re-reddening” phenomenon of the steel plate appeared with time, and the measuring point temperature increased by 37%. Second, the maximum calculated difference between the surface temperature and the measuring point temperature was 0.75 °C, and the integrated heat transfer coefficient conformed to the periodic boundary features. The comprehensive convective heat transfer coefficient on the surface was in agreement with the periodic boundary characteristics, and its value exhibited oscillatory attenuation with the cooling process, and the oscillatory peak period was about 6 seconds. Two methods, sequential function method (SFM) and finite difference method (FDM), were used to verify the correctness of TSFM.

## 1. Introduction

Temperature control in the plate cooling process has always been very complex. It is related to the cooling medium’s flow rate, velocity, pressure, and other process parameters and the specific heat transfer mechanism, such as phase change heat transfer, convection heat transfer, radiation heat transfer, and heat transfer conduction. By controlling the cooling temperature and cooling rate, the transformation process of austenite grains and the mechanical properties of steel can be improved [1].

Liu et al. [2] first adopted numerical simulation using COMSOL software to calculate the temperature field of the plate in the laminar cooling process. Next, the experimental scheme was determined according to the calculated temperature field. Subsequently, holes were drilled at different positions along the slab thickness direction, and the temperature curve of the slab in the cooling process was measured by using the “buried couple method”. Finally, the temperature gradient was obtained, and the convection heat transfer coefficient was calculated. The obtained results showed that: (1) the time required for uniform cooling of the whole plate was proportional to the plate thickness; and (2) the convection heat transfer coefficient increased with the water flow. Serajzadeh [3] investigated various properties of the steel plate in the cooling process. He coupled the dynamic equation of phase change with the differential equation of heat conduction and established a mathematical model that could predict temperature, the degree of phase change, and other variables. Then, the cooling curve of the steel plate and the pearlite and ferrite productivity of the slab surface and core were calculated and analyzed under air cooling and quenching conditions. Finally, the reliability of the model was verified using the laminar cooling experiments and metallographic observations. Chen et al. [4] used similar methods to discuss the change of physical parameters, such as thermal conductivity and specific heat capacity, with different carbon contents and temperature and compared the results with the actual production data of Baosteel to improve the model accuracy. From the perspective of process parameters and cooling medium properties, Lily et al. [5] experimentally analyzed the influence of the nozzle-to-slab angle, cooling medium viscosity, surface tension, and other parameters on the heat transfer process by selecting three different surfactants. The results showed that reducing the surface tension and viscosity of the cooling medium could reduce the residence time of the cooling medium on the slab surface and significantly improve the heat exchange efficiency. In addition, reducing the relative angle of the nozzle enhanced the wetting degree of the slab by the cooling medium and improved the heat exchange efficiency. Yu et al. [6] established an experimental system with R22 as a cooling medium. During the cooling process, the convection heat transfer coefficient, cooling surface temperature, critical heat flux, and other characteristics of the system were studied in the range of 0.6–1.0 MPa nozzle pressure. The obtained results indicated that the heat flux density increased and reached the peak value at 0.34 MPa with the nozzle outlet pressure.

Based on the microstructural changes, the coupling of austenite transformation kinetics and heat transfer was considered in the above research. The laminar cooling heat transfer mainly involved boiling heat transfer, convection heat transfer, radiation heat transfer, and heat conduction. However, there are only a few studies discussing the process factors, such as cooling medium temperature, flow rate, flow velocity, and the height and the inclination angle of the nozzle.

To sum up, many factors may affect the laminar cooling efficiency, as shown in Table 1.

The laminar cooling device and the used method are important parts of the hot rolling lines in the iron and steel industry. Regarding temperature control, a header or a water curtain is used to control flow parameters of the cooling medium, while spray quenching and cooling are used for the medium-thick slab.

However, the precise control model of temperature is derived by mathematical description and boundary conditions. Yet, only a few studies on the characteristics of boundary conditions are available in the literature, mainly due to the following reasons: it is difficult to perform industrial-scale experiments in the laboratory, and boundary conditions are discontinuous during the cooling process, so the boundary information is isolated and fragmented, and the temperature distributions of the medium and heavy slabs are difficult to measure. The problem based on the above boundary conditions is called the inverse heat transfer problem (IHTP) [24]. It was extensively studied [25,26,27,28] but rarely applied in industrial experiments.

In this paper, the variation of the slab cross-section temperature was discussed on an industrial-grade laminar cooling experimental platform. The time series function method (TSFM) was used to solve the IHTP of the one-dimensional temperature field in the laminar cooling process. The surface temperature of the slab and the comprehensive convective heat transfer coefficient (boundary conditions of the third kind) were calculated. Finally, accurate control of the laminar cooling temperature of the medium and thick slab is shown to be very significant.

## 2. Experiment

### 2.1. Experimental Raw Material

A Q235B steel plate with a size of 300 mm × 300 mm × 35 mm was used as an experimental material, and water at 25 °C was used as a cooling medium.

### 2.2. Experimental Apparatus

**Figure 1:**
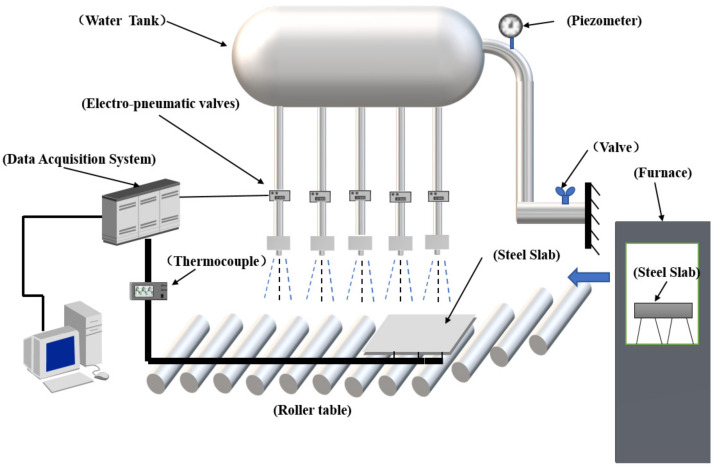
Schematic diagram of the laminar cooling experimental platform.

The laminar cooling experimental platform designed and built by our research group was used. As shown in Figure 1, the experimental setup was mainly composed of heating, cooling, and control systems. The cooling system included four parts: water tank, flowmeter, header nozzle, and moving roller table.

The header nozzle can be applied to all cooling media, with an accurate and controllable flow and a wide application range. Ten pipes were adopted, and their two ends were fixed with shelves. The top end had an inlet. The flowmeter was connected to the inlet by a hose so that the distance and height of the pipes could be adjusted. The nozzles were distributed along the axial direction of the bottom of the pipes. The number of nozzles on each pipe was 10, for a total of 100, the nozzle diameter was 3 mm, and the distance was 5 mm. The nozzle distribution corresponded to the width of the moving roller table below to prevent water splashing out.

The motor controlled the moving roller table, and the moving speed was adjustable in the range from 1 to 5 m/min.

### 2.3. Experimental Method

**Figure 2:**
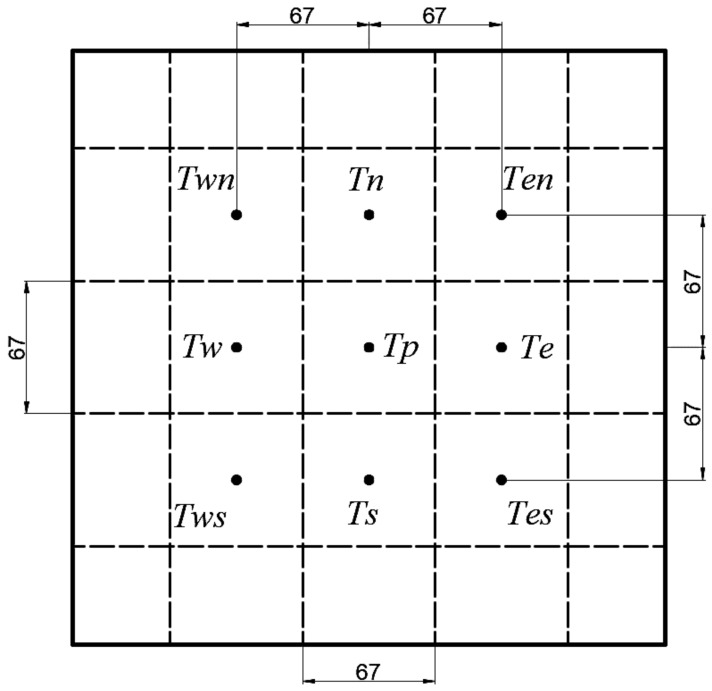
Steel plate hole location map.

Holes were drilled at the bottom of the Q235B steel plate, with a hole diameter of 5.5 mm, a drilling depth of 34 mm, and a distance from the upper surface of 1 mm. The hole location is shown in Figure 2. The thermocouple was embedded in the bottom of the hole, and the pores were filled with insulating cotton. The diameter of the thermocouple wire was 0.5 mm, and the length was longer than that of the roller table. The thermocouple penetrated a protective sleeve composed of several insulating ceramic materials with an outer diameter of 2 mm and an inner diameter of 1 mm, which prevented the thermocouple from breaking or becoming entangled as it moves along the roller table. The steel plate was placed in the heating furnace for heating and heat preservation. When the temperature was even to 800 °C, it was taken out of the heating furnace and placed at the starting point of the moving roller table. Next, the speed of the moving roller table was adjusted, and the nozzle spraying switch was turned on to cool the steel plate at a fixed water flow rate. Finally, the data were collected by the control system and analyzed. The experimental conditions are shown in Table 2. In this experiment, the accuracy of the results can be calculated by the following formula [29]:

δU=∑i=1J∂U∂XiδXi212

where *U* = *U* (*X*_1_, *X*_2_, *X*_3_, ···, *X*_J_) refers to the accuracy of the experimental results. *X* refers to the experimental variables, and ${\delta X}_{i}$ refers to the accuracy of each experimental variable, as shown in Table 3.

## 3. Experimental Results and Analysis

### 3.1. The Rule of Temperature Changing with Time at the Tp Position in the Whole Cooling Process

**Figure 3:**
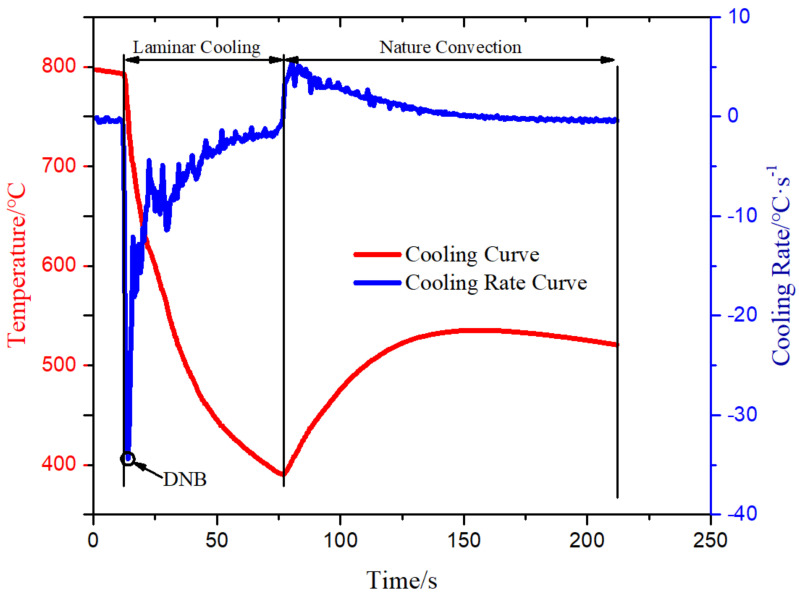
Curves of the cooling temperature and the temperature rate for *Tp.*

Figure 3 shows the temperature change and the temperature drop rate with time at the *Tp* position when the water flow was 0.125 m^3^/h and the steel plate running speed was 3 m/min. When the flow rate and the running speed did not change, the steel plate was taken out of the heating furnace and placed on the moving roller table within 0–12.5 s, which belongs to the natural convection heat exchange between the surface and the air. The temperature at *Tp* decreased from 797.1 to 793.2 °C, which was a decrease ratio of 0.49%. The temperature drop rate fluctuated and declined, having an average value of −0.36818 °C/s. The main reason was that the plane where the temperature measuring point was located was very close to the surface. The surface involved the natural convection heat transfer of the air and the internal heat conduction of the steel plate, so the heat transfer rate was unbalanced. From 12.5 to 77 s, the steel plate ran on the moving roller table, and the nozzle sprayed cooling water for laminar cooling. From 12.5 to 14 s, the temperature and the cooling rate at the temperature measuring point exhibited a sharp change. The temperature dropped from 793.2 to 730 °C, with a drop ratio of 8%, and the cooling rate decreased from −0.9 to −34 °C/s, with a drop ratio of 36%. This was caused by the intensive convection boiling heat transfer between cooling water and the surface of high-temperature steel plate, disturbing each other, then transferring toward the inside of the steel plate, and interrupting the internal heat conduction of the steel plate again. It was suggested that the nucleate boiling on the surface of the steel plate occurred suddenly in this timeframe, so the departure nucleate boiling point (DNB) was at 14 s. From 14 s, the value of temperature drop rate started to increase, but the increasing trend fluctuated, and the temperature drop rate slowed down. From 14 to 44 s, the temperature drop rate greatly fluctuated, representing a transitional boiling region. At 44 s, the numerical fluctuation amplitude of the temperature drop rate attenuated and became stable film boiling between 44 and 155 s.

Based on the analysis of the experimental phenomena mentioned above, the main causes of the temperature fluctuation are summarized as follows: (1) when cooling water impinges on the surface of the steel plate, a vapor film is formed on the surface under the action of high temperature, which is equivalent to thermal resistance; the film thickness will change with the impact of cooling water, so the film is unstable; (2) the heat transfer from the interior to the surface of the steel plate requires a certain period; (3) the latent heat of crystallization is released during the process of austenite transformation.

At 77 s, the laminar cooling was over. There was still cooling water covering the surface of the steel plate, and the internal heat conduction rate of the steel plate was greater than the convection heat exchange rate of the surface, so the temperature at the measuring point started to increase, and the “re-reddening” phenomenon appeared. In the timeframe between 77 and 155 s, the temperature rose from 395 to 535 °C, exhibiting an increase of 35%. From 155 s, the evaporation of cooling water on the surface of the steel plate finished, and the natural convection heat transfer process between the steel plate and the air resumed.

### 3.2. The Temperature Change with Time at Other Positions in the Whole Cooling Process

**Figure 4:**
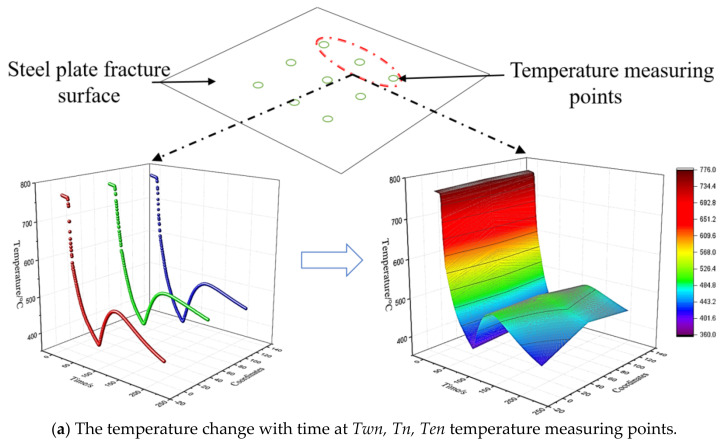

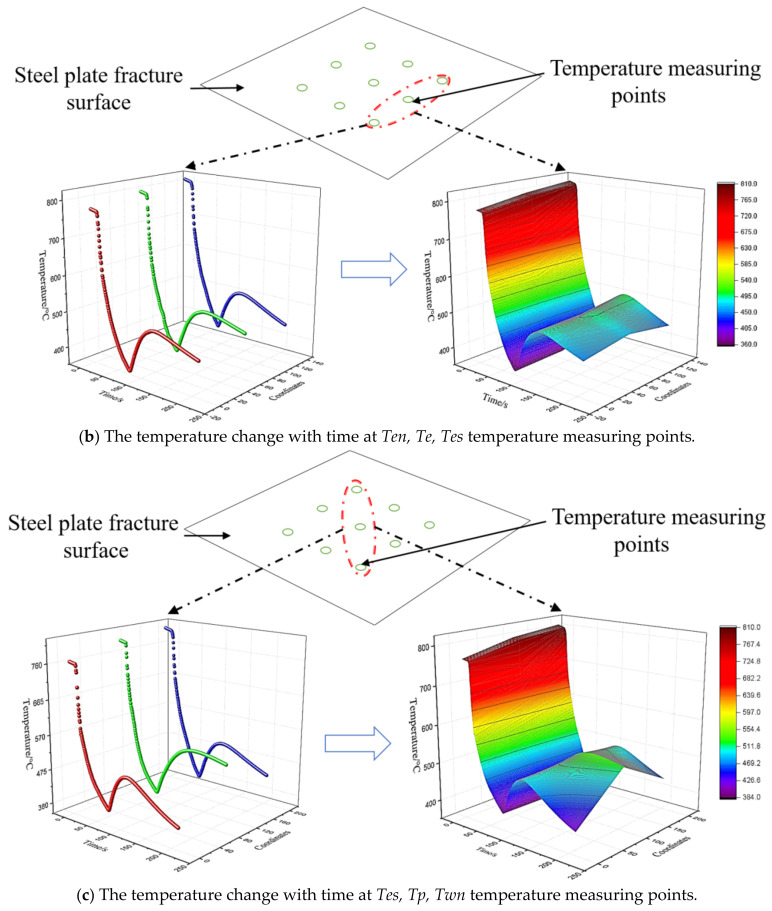
The temperature distribution rule of the three groups of temperature measuring points with time.

Nine temperature measuring points (*Twn, Tn, Ten*) (*Ten, Te, Tes*) (*Tes, Tp, Twn*) were taken at the section position 1 mm from the surface and divided into three groups. Then, the temperature change and the temperature drop rate with time at each group of temperature measuring points were analyzed. As shown in Figure 4a–c, the change trends of the three data groups were the same as that at the *Tp* position. In the period of 12.5–14 s, the temperature distribution was discontinuous with the time gradient. The main reason is that the temperature drop rate is too large, yielding a mismatch between the response time and the thermocouple response rate. By combining the three temperature measuring points of each group, it was found that the spatial position of each point is different. As can be seen from the diagram, the temperature change and the time distribution of each group of different temperature measuring points were basically the same, but the temperature at spatially different measuring points will be slightly different in a certain interval, mainly in the laminar cooling process, film boiling, transition boiling, and nucleate boiling occurred at different positions on the surface of the steel plate [30]. As the steel plate moved on the roller, water periodically washed over the steel plate, resulting in the periodic coupling of these three boiling phenomena on the steel plate surface; therefore, the surface temperature of the steel plate fluctuated periodically, and the surface temperature reflected the measuring point of the cross-section, leading to the experimental phenomenon.

## 4. Solution of Temperature and Integrated Convection Heat Transfer at the Center of the Surface by TSFM

The sequential function method (SFM) [31] was used to estimate the time order. When using this method to identify the heat flow *q*(*t*), the problem is described as follows: when it is known that the heat flow *q*_1_, *q*_2_, ..., *q_k−_*_1_ at the first *k* − 1 time and the temperature values *T_k_*, *T_k+_*_1_,..., *T_k+n−_*_1_ at the last n time, the heat flow *q_k_* at *t_k_* needs to be identified. However, this method mainly focuses on algorithm analysis, and it has not been applied in experiments and engineering [32,33]. Based on the above experimental data, this paper optimized the method and considered that the sufficient condition for the temperature change with time is heat transfer along the space direction with time. Thus, the temperature change per time unit is caused by the heat transfer in the previous period, so the third type of boundary conditions can be obtained by the layer by layer recursion. This method is called the time sequence function method (TSFM).

### 4.1. Solution of Temperature at the Center of the Laminar Cooling Surface

According to the above problem description, the temperature distribution with time at the temperature measuring point 1 mm from the surface was experimentally obtained. Based on the experimental conditions and results, the problem is summarized by using the TSFM as follows:

(1) The temperature distribution of the steel plate is mainly along its thickness direction, so the cooling process is simplified as a one-dimensional unsteady heat transfer process, that is, from *Tp* to the center of the steel plate surface, the thickness is 1 mm;
(2) The time and space are dispersed from the *Tp* position to the center of the steel plate surface. The time step is ∆*t* = 0.5 s according to the thermocouple response time. The calculation time is 12.5–77 s, i.e., the laminar spray cooling process, yielding *N* = 54/∆*t* = 109;
(3) The heat transfer process of the steel plate includes only the heat conduction and the surface convection heat transfer, ignoring the radiation heat transfer;
(4) There is no internal heat source;
(5) The bottom of *Tp* is filled with cotton, which can be considered as insulation. The heat is transmitted along the direction perpendicular to the upper surface, so the temperature change at each time step is caused by the temperature difference in the center of the surface, namely:

$${\rho c}_{p}\frac{T_{p}^{t_{1}}-t_{p}^{t_{2}}}{\Delta t}=\lambda \frac{t_{p}^{t_{1}}-t^{t_{1}}}{\Delta X}\;{\rho c}_{p}\frac{t_{p}^{t_{2}}-t_{p}^{t_{3}}}{\Delta t}=\lambda \frac{t_{p}^{t_{2}}-t^{t_{2}}}{\Delta X}\;{\rho c}_{p}\frac{t_{p}^{t_{3}}-t_{p}^{t_{4}}}{\Delta t}=\lambda \frac{t_{p}^{t_{3}}-t^{t_{3}}}{\Delta X}\;\vdots \;{\rho c}_{p}\frac{t_{p}^{t_{N-1}}-t_{p}^{t_{N}}}{\Delta t}=\lambda \frac{t_{p}^{t_{N-1}}-t^{t_{N-1}}}{\Delta X}$$

where:

ρ—Density of the steel plate (kg/m^3^);

λ—Thermal conductivity of the steel plate (W/(m·K));

$c_{p}$—Specific heat capacity of the steel plate (J/(kg·K));

The thermophysical parameters of the steel plate are only a function of temperature [4], then:

$$\left\{\begin{array}{c} \lambda =-0.0335t+50.4726,t\in (0\text{–}790);\\ \lambda =0.0058t+17.521,t\in (790\text{–}1000); \end{array}\right.\;\left\{\begin{array}{c} c_{p}=0.8613t+254.8,t\in (0\text{–}800);\\ c_{P}=647.56,\;t\in (800\text{–}1000); \end{array}\right.$$

**Figure 5:**
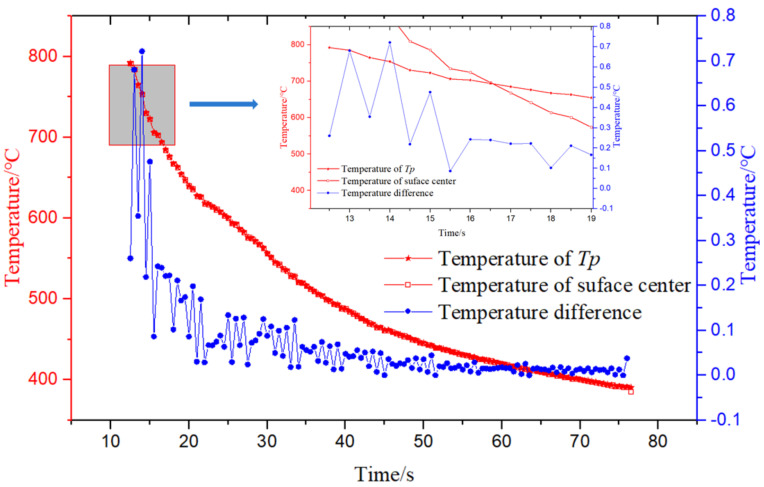
Distribution of temperature and temperature difference with time at the surface center and *Tp.*

(1) The heat transfer process of the steel plate includes only the heat conduction and the surface convection heat transfer, ignoring the radiation heat transfer;
(2) There is no internal heat source;

According to the above mathematical method, the temperature distribution with time at the center of the steel plate surface was calculated. Figure 5 represents the temperature distribution curve with time at the center of the surface and at *Tp* obtained from the experiment from the beginning of laminar cooling, i.e., 12.5–76.5 s. The temperature value and the changing trend at the surface position and *Tp* were basically the same. At 12.5–14 s the laminar cooling started, and the temperature at the center and *Tp* of the steel plate surface dropped rapidly under the action of cooling water. At this time, the convection heat transfer dominated on the surface, and there was damping and delay in the heat transfer process. Therefore, the temperature difference between the center and *Tp* of the surface was large, having the maximum value of 0.75 °C, the minimum value of 0.35 °C, and the average value of 0.5 °C. With the increase in the cooling time, in the period of 14–44 s, the convection heat transfer and the boiling heat transfer processes were coupled on the surface, while the gas film was gradually generated and became thicker with the transition from nuclear boiling to film boiling, so the temperature difference value fluctuated and decreased under the disturbance of the two heat transfer modes. After 44 s, there was a stable film boiling heat transfer. The gas film was relatively stable and increased the heat transfer resistance. The amplitude of the temperature difference value decreased and finally tended to be constant.

### 4.2. Solution of the Integrated Convection Heat Transfer at the Center of the Laminar Cooling Surface

**Figure 6:**
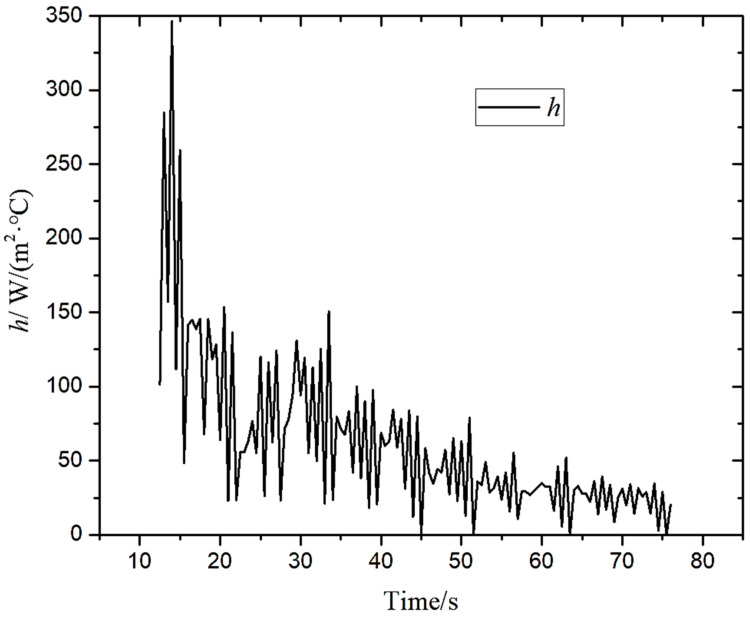
The change of the integrated convection heat transfer coefficient with time at the surface center.

According to the temperature value at the center of the surface and the corresponding boundary conditions, the integrated convection heat transfer coefficient generated by the coupling of the boiling heat transfer and the convection heat transfer at this position was solved by Newton cooling formula. Figure 6 shows the variation of integrated convection heat transfer coefficient *h* with time at the center of the surface. The *h* value changed periodically with time in the laminar cooling process. The main reasons are as follows: (1) the steel plate runs at a speed of 3 m/min on the roller table, and close to it, there are discharge nozzles in the vertical direction of the running speed to spray water on the steel plate for cooling, so the water quantity covering the center conforms to the periodic change; (2) the laminar cooling process is a coupled process of boiling and convection heat transfers, in which the gas film formation and growth occur, while the periodic fluctuation occurs under the action of water flow disturbance. As shown in Figure 6, at 14 s, *h* reached the maximum value of 345 W/(m^2^·°C); from 14 to 44 s, it decreased by 74%; from 44 to 76.5 s, the *h* value stabilized at about 44 W/(m^2^·°C). From the calculation results, the fluctuations of the value of the comprehensive convective heat transfer coefficient and the temperature difference were almost constant, and the temperature difference exerted a very strong influence on them.

### 4.3. Solution of the Temperature and the Integrated Convection Heat Transfer Coefficient at Other Positions of the Laminar Cooling Surface

**Figure 7:**
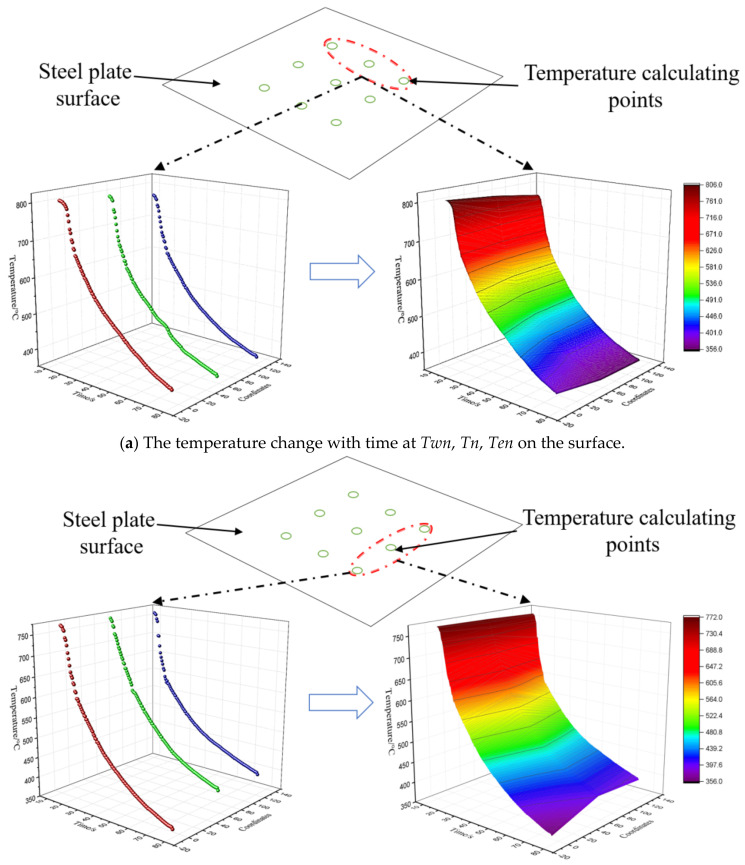

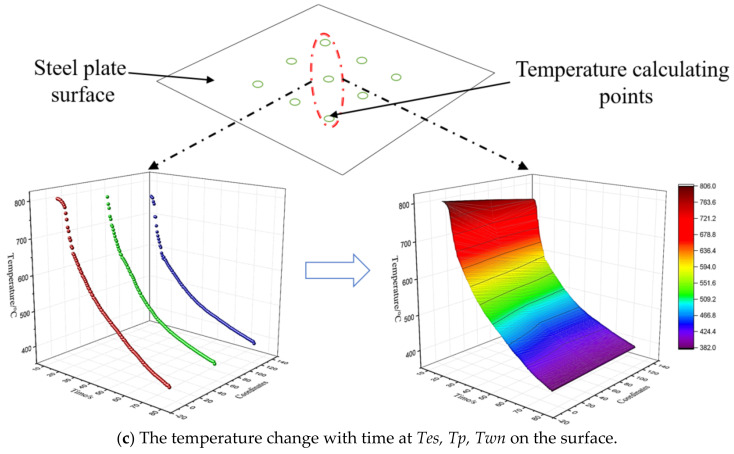
The temperature change with time at different points on the surface.

The same method was adopted to calculate the temperature and the integrated convection heat transfer coefficient of *Twn*, *Tn*, *Ten*, *Te,* and *Tes* located on the surface during the laminar cooling process. The results are shown in Figure 7a–c. The change of surface temperature with time was almost the same as that at the section, i.e., the temperature reduction rate firstly increased and then decreased with the cooling time. The maximum temperature difference at 1 mm from the surface was 0.8 °C, the minimum temperature difference was 0 °C, and the fluctuation range was very small. The results show that the heat conduction process between the section 1 mm away from the surface and the steel plate surface agrees with the condition of the lumped parameter method, which can be simplified to a zero-dimensional process.

**Figure 8:**
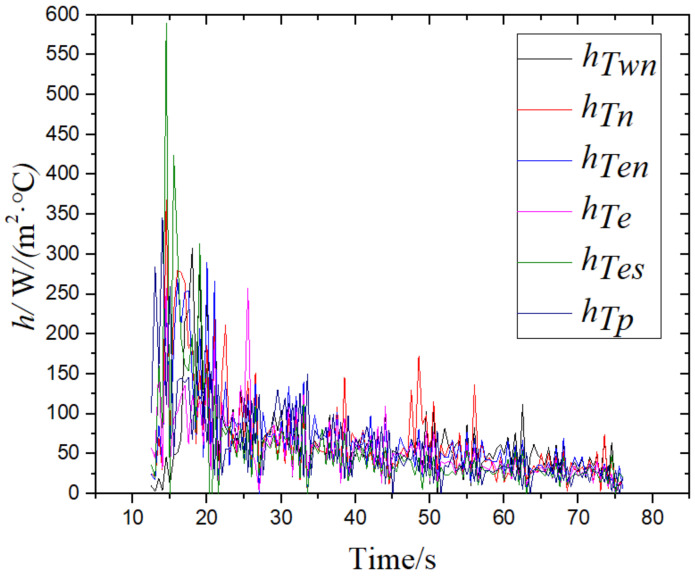
The change of the convection heat transfer coefficient with time at different points on the surface.

**Figure 9:**
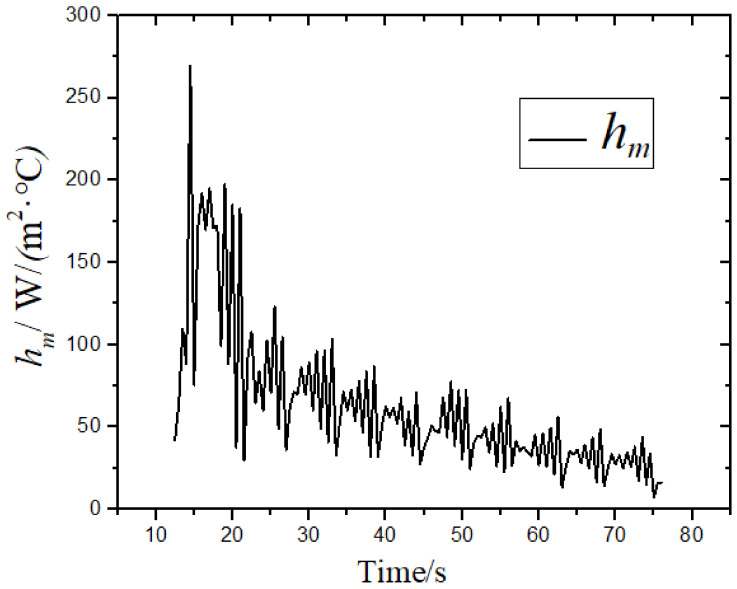
The change of the average surface integrated convection heat transfer coefficient with time.

As shown in Figure 8, according to the change of the integrated convection heat transfer coefficient with time, the difference in the *h* value at each point was also small, and the change of the surface average integrated convection heat transfer coefficient with the laminar cooling time was obtained after the data are sorted, as shown in Figure 9.

Figure 9 shows that the heat transfer characteristics in the laminar cooling process essentially conformed to the periodic heat transfer boundary characteristics; that is, the boundary condition *h* value of the heat transfer process had obvious periodic characteristics. The size range of the *h* value was consistent with the literature data [34,35].

### 4.4. Verification of the Calculation Results

In this paper, two methods (SFM and FDM) were used respectively to verify the TSFM calculation results directly and indirectly. SFM directly compared the calculated results with TSFM; The results of TSFM were brought into the recalculation by FDM and compared with the experimental results.

#### 4.4.1. Verification Using SFM

In this paper, the sequential function method (SFM) was used to verify the results calculated by TSFM. The SFM uses a finite volume method and an alternate implicit algorithm to find the rate of temperature change per unit of heat, called the sensitivity coefficient, and solves the surface heat flux distribution according to the sensitivity coefficient and the calculated temperature difference. According to the corresponding heat flux, the surface temperature and the comprehensive convective heat transfer coefficient are solved by a mathematical description as follows:

Assuming that the heat flux changes in the time interval ($t_{k},t_{k+r-1}$), this satisfies the following equation:

$$q_{k}=q_{k+1}=\ldots =q_{k+r+1}$$

where vector $q_{k}$ is defined as: $q_{k}\;$*=*$\;[q_{k}\left(1\right),q_{k}\left(2\right),\cdots ,\;q_{k}{\left(n\right)}^{T}]$, n=1,2,…,N; n is the heat flux to be retrieved at each time. N is the total inverse number.

Defining $S\left(x,t\right)=\partial T\left(x,t\right)/\left(\partial q_{k}\right)$ as a sensitivity function, this represents the sensitivity of temperature field $T\left(x,t\right)$ to heat flux $q_{k}$.

The sensitivity function $S\left(x,t\right)$ for the ($t_{k},t_{k+r-1})$ time period is obtained by derivation of $q_{k}$ from the heat differential equation. It can be found from the sensitivity equation that $S\left(x,t\right)$ is independent from $q_{k}$. Therefore, the dynamic step response coefficients can be obtained by solving the step response equations using the finite volume method and the alternating implicit algorithm.

The objective function is defined as follows:

$$G\left(q_{k}\right)=\overset{M}{\underset{m=1}{\sum }}\overset{r}{\underset{i=1}{\sum }}{\left(Y_{m,k+i-1}-T_{m,k+i-1}\left(q_{k}\right)\right)}^{2}$$

$Y_{m,k+i-1}$ and $T_{m,k+i-1}\left(q_{k}\right)$ in the formula are the temperature values measured and calculated at the point m at time $t_{k+r-1}$, respectively.

By applying the calculated temperature at point m to Taylor’s expansion at the point $q_{k}=q_{k}^{0}$, we obtain:

$$\;\;\;\;\;\;\;\;\;\;\;\;\;T_{m,k+i-1}\left(q_{k}\right)=T_{m,k+i-1}\left(q_{k}^{0}\right)+\overset{N}{\underset{n=1}{\sum }}S_{m,n,i}\left(q_{k}\left(n\right)-q_{k}\left(n\right)\right)$$

In the formula, $S_{m,n,i}=\partial T_{m,k+i-1}/\partial q_{k}\left(n\right)$ represents the temperature sensitivity coefficient under the disturbance of a unit heat flow.

Combining the two formulas and letting $\partial G\left(q_{k}\right)/\partial q_{k}\left(n\right)=0$, we obtain:

$$2\overset{M}{\underset{m=1}{\sum }}\overset{r}{\underset{i=1}{\sum }}\left[T_{m,k+i-1}\left(q_{k}^{0}\right)-Y_{m,k+i-1}+\overset{M}{\underset{m=1}{\sum }}S_{m,n,i}\left(q_{k}\left(n\right)-q_{k}^{0}\left(n\right)\right)\right]S_{m,n,i}=0\;\;$$

In the last equation, m=1,2,…,M;n=1,2,…,N;i=1,2,…,r.

By expanding the above formula, we can obtain the solution expression of $q_{k}$, which is integrated into a matrix form as follows:

$$q_{k}=q_{k}^{0}+{\left(S^{T}S\right)}^{-1}S^{T}\left(Y-T\left(q_{k}^{0}\right)\right)$$

**Figure 10:**
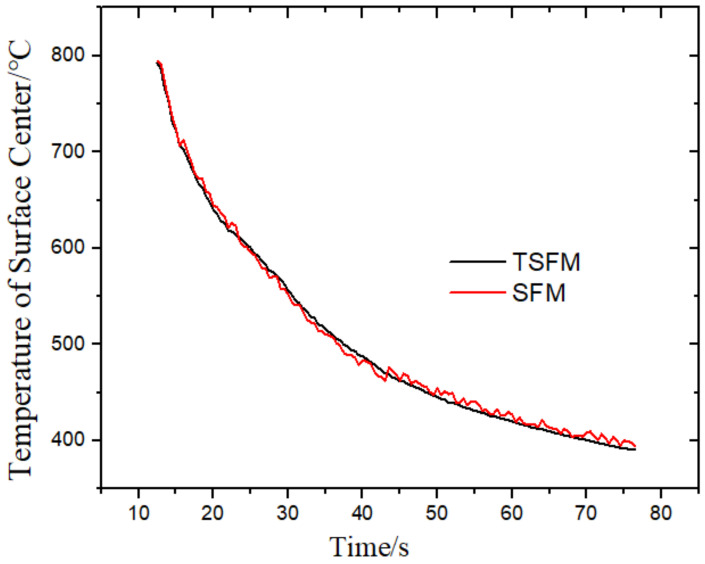
Comparison of the surface center temperature calculated by two methods.

**Figure 11:**
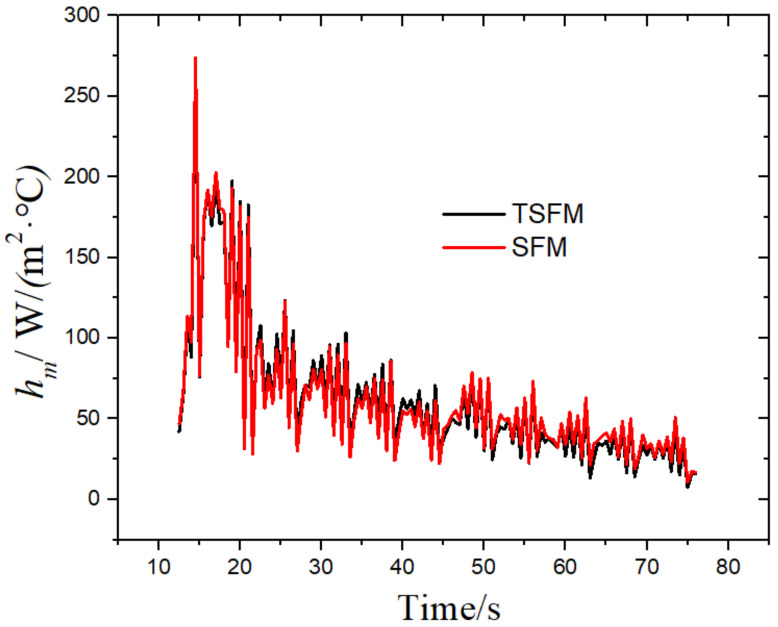
Comparison of the comprehensive convective heat transfer coefficients calculated by the two methods.

According to the heat flux, the temperature field is solved by the finite volume method and the alternate implicit method, and the surface temperature and comprehensive convective heat transfer coefficient are obtained. The results are shown in Figure 10 and Figure 11. The calculation results of the two methods were very similar. The relative mean deviation of temperature was 0.39%, and the relative mean deviation of the comprehensive convective heat transfer coefficient was 0.18%, which fully proves the feasibility of TSFM. The TSFM uses the thermocouple response speed in the experiment as the time step, eliminating the influence of the future time step in the SFM on the calculation results and facilitating industrial applications.

#### 4.4.2. Verification Using FDM

In order to prevent accidental occurrence in the verification process, this paper used the mature method of finite difference method (FDM) [36] to bring the boundary conditions calculated in TSFM into FDM and compared the calculated results with the experimental results, so as to indirectly verify the correctness of TSFM based on the experimental data, as follows: 

For the one-dimensional plate and unsteady heat conduction problem, the governing equation is as follows:

$$\rho C\frac{\partial T}{\partial t}=\frac{\partial }{\partial x}\left[\lambda \frac{\partial T}{\partial x}\right]$$

TSFM calculation results were selected as the boundary conditions:

$$B_{1}:\;\frac{\partial T}{\partial x}=o;x=0\;B_{2}:-\lambda \frac{\partial T}{\partial x}=h\left(T-T_{\infty }\right);x=\delta$$

**Figure 12:**
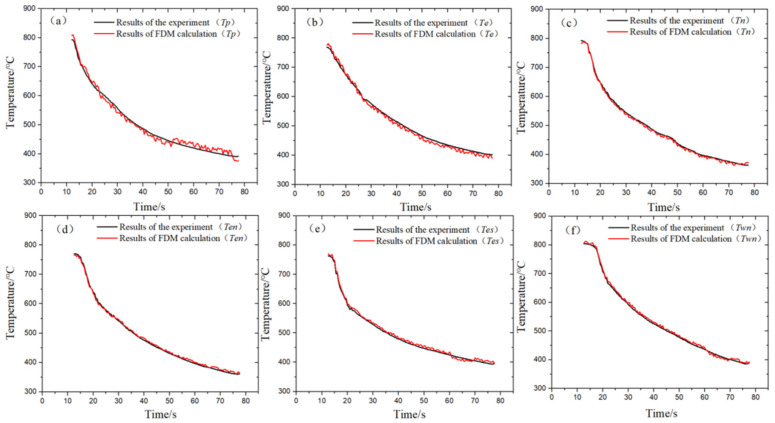
FDM calculated temperature and experimental temperature comparison. (**a**) *Tp* position experiment and simulation comparison, (**b**) *Te* position experiment and simulation comparison, (**c**) *Tn* position experiment and simulation comparison, (**d**) *Ten* position experiment and simulation comparison, (**e**) *Tes* position experiment and simulation comparison, (**f**) *Twn* position experiment and simulation comparison.

The FDM was used to discretize the solution area, and the Tri-diagonal matrix algorithm (TDMA) [37] was used to solve the discrete equations. Six temperature measurement points such as *Tp* were calculated. The calculation results are shown in Figure 12. The calculated temperature was basically consistent with the experimental temperature, and the relative experimental average error range of the six points was [−2.8–4.9%]. The reason may be because of the reduction from three dimensions to one. We will continue to pay attention to more multidimensional inversion simulation in subsequent studies.

## 5. Conclusions

In this paper, the “buried couple method” was used to measure the temperature value of the temperature measuring point on the cross-section 1 mm from the surface. The sequential function method was used to calculate the corresponding point temperature and the integrated convection heat transfer coefficient on the surface. The obtained results yielded the following points:

(1) At the beginning of water cooling, the temperature of the measuring point dropped sharply, with a drop rate of 8% and a drop multiplying power of 36%; at the stage of air cooling, the temperature of the measuring point ascended, with an increase of 35%, showing the phenomenon of “re-reddening”.
(2) In the laminar cooling process of the hot-rolled steel plate, the surface temperature and the integrated convection heat transfer coefficient of the surface exhibited non-linear behavior with time. When the cooling time was 12.5–14 s, the surface temperature decreased sharply, while the integrated convection heat transfer coefficient increased periodically with time. When the time was more than 14 s, the surface temperature dropping rate decreased, and the integrated convection heat transfer coefficient declined periodically, finally approaching slight changes;
(3) The SFM was used to verify the calculated results. The relative average errors of the surface temperature and the comprehensive convective heat transfer coefficient were 0.39 and 0.18%, respectively, which proved that the TSFM was a successful approach.
(4) The FDM was used to verify the correctness of the calculated results indirectly. The results show that when the boundary conditions calculated by TSFM were brought back to the governing equation for solving, the calculated results were in good agreement with the experimental results.

The above results indicate that in the laminar cooling time range, the integrated convection heat transfer coefficient of the steel plate surface conforms to the periodic boundary characteristics, which provides a scientific basis for the subsequent study of the influence of the periodic boundary characteristic parameters on the billet temperature field, the disclosure of the coupling mechanism of the heat transfer boundary characteristics and the heat transfer process, and the structure and performance of the billet.

## Figures and Tables

**Table 1:** Factors that influence the laminar cooling efficiency.

Types	Process Parameters	Fluid Properties	Steel Plate Properties	Environmental Properties
	Nozzle types [7]	Thermal properties of fluid [8]	Thermal properties of steel plate [9]	Air properties [10]
	Nozzle height [11]	Fluid viscosity [12]	Surface roughness [13]	
	Nozzle caliber [14]	Surface tension [15]	Undercooling [16]	
	Jet volocity [17]	Surfactant [18]		
	Nozzle angle [19]	Latent heat of vaporization [20]		
	Jet flow rate [21]			
	Fluid temperature [22]			
	Droplet size [23]			

**Table 2:** Experimental conditions at 1 mm from the hole to the upper surface.

Experimental Condition	Water Temperature	Nozzle Height	Nozzle Diameter	Nozzle Water Flow	Steel Plate Speed
Case1	25 °C	465 mm	3 mm	0.125 m^3^/h	3 m/min

**Table 3:** Uncertainties in the experimental parameters.

Experimental Parameter	Uncertainty
Thermocouple response time/s	±0.01
Plate speed/m/min	±0.03
Water temperture/°C	±0.5
Nozzle height/mm	±0.1
Water flow/m^3^/h	±0.001
Thermocouple accuracy/°C	±0.01
